# Mitigation of Effects of Occlusion on Object Recognition with Deep Neural Networks through Low-Level Image Completion

**DOI:** 10.1155/2016/6425257

**Published:** 2016-06-01

**Authors:** Benjamin Chandler, Ennio Mingolla

**Affiliations:** ^1^Hewlett Packard Labs, Hewlett Packard Enterprise, 3000 Hanover Street, Palo Alto, CA 94304-1112, USA; ^2^Department of Communication Sciences and Disorders, Northeastern University, 360 Huntington Avenue FR226, Boston, MA 02115, USA

## Abstract

Heavily occluded objects are more difficult for classification algorithms to identify correctly than unoccluded objects. This effect is rare and thus hard to measure with datasets like ImageNet and PASCAL VOC, however, owing to biases in human-generated image pose selection. We introduce a dataset that emphasizes occlusion and additions to a standard convolutional neural network aimed at increasing invariance to occlusion. An unmodified convolutional neural network trained and tested on the new dataset rapidly degrades to chance-level accuracy as occlusion increases. Training with occluded data slows this decline but still yields poor performance with high occlusion. Integrating novel preprocessing stages to segment the input and inpaint occlusions is an effective mitigation. A convolutional network so modified is nearly as effective with more than 81% of pixels occluded as it is with no occlusion. Such a network is also more accurate on unoccluded images than an otherwise identical network that has been trained with only unoccluded images. These results depend on successful segmentation. The occlusions in our dataset are deliberately easy to segment from the figure and background. Achieving similar results on a more challenging dataset would require finding a method to split figure, background, and occluding pixels in the input.

## 1. Introduction

Objects that are heavily occluded are more difficult for a classification algorithm than unobstructed objects. We introduce a novel method for mitigating the difficulty of classification through dataset augmentation via inpainting, which in turn relies on the ability to segment object pixels from background or occluder pixels. Adaptive filters and neural networks (multilevel perceptrons) have been previously applied successfully to video sequences with occlusion in optical flow algorithms for 2D object tracking [1]. This approach has been extended to stereoscopic video streams [2]. The focus of the present work is instead on the effects of occlusion on recognition accuracy in classification of static images.

Our research is motivated by a significant divergence between human vision and machine vision in dealing with occluded objects. The human visual system excels at recognizing objects in our environment that are partially or significantly occluded by intervening surfaces. Comparable performance for computer vision has yet to be demonstrated. See [3, 4] for reviews of extensive literature on the neuroscience of human vision as related to perception of occluded objects. Notably discussed in [3, 4] are observations in [5, 6] of situations where the appearance of visible occluders can improve human performance as compared to viewing of the same fragments of objects depicted in the same spatial arrangement but in isolation, without the contours of occluding surfaces being denoted. Our research is a step in closing the gap in performance between humans and machines for recognition of occluded objects.

Neither of the current high-profile object classification benchmarks is well-suited to quantify the degree of difficulty to classification posed by variable levels of occlusion [7, 8]. Both were constructed from public photo-sharing sites and thus have a bias against occluded images. Hoiem et al. [9] used results from the 2007 PASCAL VOC classification task to identify the most significant sources of error. Classification performance decreased precipitously as the percentage of occluded pixels increased. The infrequent occurrence of occlusion in the PASCAL data, however, caused the authors to conclude that occlusion resilience has a negligible impact on the total PASCAL performance of an algorithm [9].

This assertion follows from the source of the data. Humans captured the images in PASCAL by deliberately composing and capturing scenes and as such* tended to take photographs of unobstructed objects close to the center of the frame*. This tendency towards fully visible objects in PASCAL is an example of observer bias in dataset construction [10]. Dataset bias in general, of which observer bias is one subclass, allows algorithms to produce impressive-looking classification results by overrelying on signals that have a little real-world utility. A specific dataset, for instance, might contain objects in only a very restricted set of poses or image backgrounds that strongly correlate with the class of the target object [10, 11]. This bias is difficult to measure and difficult to correct, weakening the argument that the current large classification datasets accurately represent the real world [10].

Besides representing a biased sample of the real world, the current large scale datasets are also missing much of the descriptive metadata needed to accurately quantify occlusion robustness. An ideal dataset would include a precise description of the type and level of occlusion as metadata with each sample. Hoiem et al. [9] were only able to consider four different cases for occlusion: none, low, medium, and high. The study defined low as “slight occlusion,” medium as “significant part is occluded,” and high as “many parts missing or 75% occluded.” The coarse definitions of occlusion followed from the nature of the data. Everingham et al. [8] constructed PASCAL by aggregating and hand-annotating a large collection of images from the Flickr photo-hosting website. Hoiem et al. [9] enhanced the PASCAL annotations with additional detail on level of occlusion. As in Everingham et al. [8], they had to do this by hand. This is a standard problem with nonparametric classification datasets. Such datasets are collected from unlabeled, unconstrained image sources. The data is thus very diverse, but annotations have to be added by hand and tend to be information-poor as a practical consequence [7, 8].

**Figure 1:**
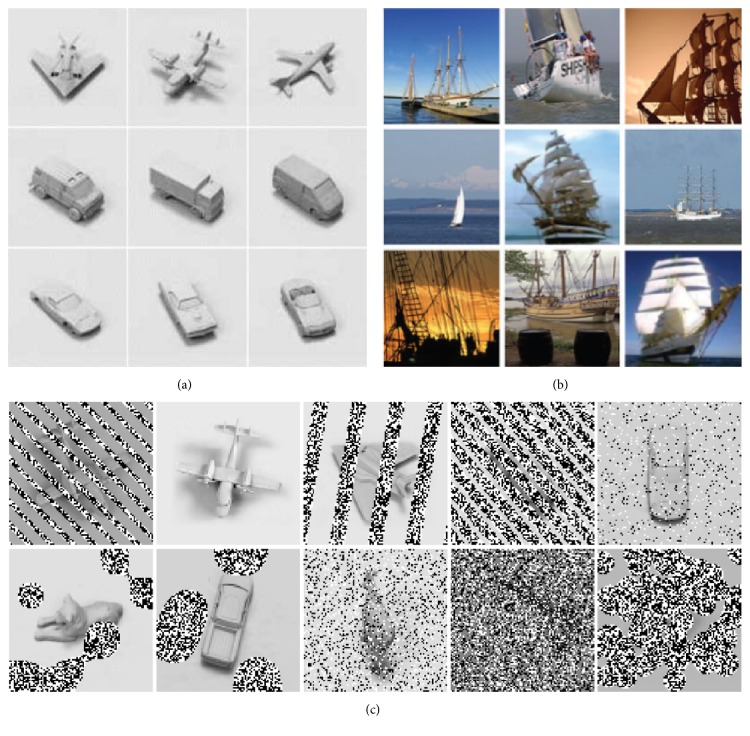
Sample images from NORB, ImageNet, and SORBO datasets. NORB (a) is a parametric dataset designed for experiments in invariant object classification. It includes five categories of objects, each with ten specific objects. NORB contains a stereo pair at nine camera elevations, eighteen camera azimuths, and five light levels for each instance [12]. ImageNet (b) is a nonparametric dataset containing a large number of labeled examples scraped from the Internet. In comparison to NORB, ImageNet has much more data and many more categories but typically only a single image for each object instance. The image parameters are unknown except for the category [7]. SORBO (c) is a new dataset that extends the NORB data. It preserves the rich parametric metadata from NORB but adds various levels of bar, blob, and random stereo occlusions.

The NORB dataset is a contrasting example of a recent parametric dataset [12]. NORB contains five image classes with ten objects in each. For each object, the dataset contains a stereo image pair for each of 36 azimuths, 9 elevations, and 6 lighting conditions. These parameters are carried along with the image data, allowing investigators to directly measure the sensitivity of their algorithms to properties like intraclass variation or lighting. Figures 1(a) and 1(b) contrast parametric and nonparametric types of classification datasets.

Performing a more sophisticated analysis of occlusion robustness than Hoiem et al. [9] were able to execute requires better descriptive metadata. This paper introduces the SORBO (Synthetic Object Recognition Benchmark with Occlusion) dataset to capture class and level of occlusion with much finer resolution than was possible by hand-annotating PASCAL data and to support work in occlusion-resistant object classification. The design matches NORB as much as possible to simplify comparison with the many NORB results in the literature.  Figure 1(c) shows example of SORBO images with varying classes and levels of occlusion.

The ability to precisely measure the impact of occlusion on object classification performance enables the construction of an occlusion-robust algorithm. Such an algorithm must confront two issues that nonrobust algorithms are able to ignore:

1. What visual information belongs to the target object?
2. How should uncertain or nontarget information be discounted?

The first problem is one of* segmentation*. An occlusion-robust classification algorithm must be able to classify each pixel of visual information as* figure*,* ground*, or* occlusion*. The figure class identifies pixels that belong to the target object. Ground pixels either are behind or do not overlap the target. Occlusions are pixels in which the object of interest is hidden because it is obstructed by an object nearer to the observer. These last two classes are distinct because occlusions cover the target and may split the figure information into two or more nonadjacent regions. The ground is either behind or nonoverlapping with the figure and thus cannot cause such splits. Once the algorithm estimates which information belongs to each class, it must then discount uncertain or nontarget information. Uncertainty arises because the segmentation of the scene is a potentially inaccurate estimate.

Segmentation and discounting are relevant tasks even for a classification algorithm, as opposed to a* detection* algorithm. In a detection problem the algorithm must estimate the location of a target object given an input image that may contain a target object at an arbitrary position in the frame. A subset of input images may contain no target objects. A* classification* problem is simpler to solve in that it enforces viewpoint bias by restricting the input to images with a single dominant object class close to the center of the frame. The Hoiem et al. [9] study indicates that occlusion is a significant issue even for classification problems.

The analysis in Hoiem et al. [9] was sufficient to establish that existing state-of-the-art algorithms do not perform well in high-occlusion scenarios. It also established that the PASCAL VOC dataset is the wrong benchmark to use if high-occlusion scenarios are important. PASCAL VOC contains relatively few occluded objects, so the aggregate results are relatively insensitive to how well an algorithm handles occlusion. For the objects that* are* occluded, the locations of the occluding pixels are unknown. This lack of ground-truth segmentation information further limits the applicability of the PASCAL VOC data to the development of occlusion-resistant object classification algorithms. SORBO includes this additional metadata.

This paper introduces a new occlusion-robust object classification algorithm that leverages the rich metadata in the SORBO dataset. This new algorithm is an* extension* of an existing algorithm rather than an entirely custom construction. Extending an existing algorithm carries the primary benefit of preserving the performance of the underlying classifier in low-occlusion cases. A state-of-the-art base classifier will continue to perform as intended on standard datasets like NORB. The new extensions allow the classifier to degrade in performance more slowly than would be the case without the algorithm extensions as occlusion increases.

Taken together, parametric occlusion metadata and ground-truth segmentation information make SORBO an ideal dataset for exploring occlusion-resistant object classification. This paper first focuses on replicating the Hoiem et al. [9] study using the new SORBO dataset. Follow-on experiments build on these results to construct an occlusion-resistant object classification algorithm from an existing nonresistant algorithm.

*Existing Object Classification Datasets*. The majority of current object classification work is organized around two standard challenge datasets: the ImageNet Large Scale Visual Recognition Challenge (ImageNet) and the PASCAL Visual Object Classes Challenge (PASCAL VOC or PASCAL). Each challenge has an associated dataset. While the two challenges use different data, different tasks, and different accuracy metrics, both are* large scale* and* nonparametric*.

In the context of object classification, a nonparametric dataset is one in which the images are sampled from a population with* unknown* image parameters. The term does not imply that the images cannot be described by a parameter space. It only implies that the investigators cannot practically enumerate the image parameters. Typically this is because the images are drawn from a very large, weakly constrained population with a very large parameter space. The ImageNet samples in Figure 1(b) illustrate this point. The images are all photographs of ships. No ship appears more than once, however, and parameters like the image composition and pose vector for the target object are all unknown. More critically, there are additional, uncontrolled parameters like the time of day, wind, and background. Simply describing all the sources of parametric variation in the images is impractical due to the size of the parameter space, the number of images, and the fact that each image requires manual labeling.

The principle difference between the PASCAL VOC and ImageNet is the number and structure of the object categories. PASCAL contains four macro categories (person, animal, vehicle, and indoor) with a total of twenty subcategories. This relatively flat category structure and low total category count is consistent with most prior object classification datasets. ImageNet differs in that it contains thousands of categories arranged in a deep, hierarchical structure. The TinyImages dataset from MIT uses a similar structure but only includes very small images [13]. Both ImageNet and TinyImages use category labels from the noun portion of the WordNet database [14]. WordNet includes superordinate and subordinate terms for each term in the database. Given the label “German shepherd,” for instance, the superordinate label list is {shepherd dog, working dog, dog, canine, carnivore, placental mammal, vertebrate, chordate, animal}. Each step up the tree of superordinate labels represents an is-a relationship. Algorithms designed for a small, flat hierarchy of category labels tend not to do well with the large, deep, hierarchical tree of category labels in the ImageNet and TinyImages datasets without modification. All three datasets (PASCAL, ImageNet, and TinyImages) are still nonparametric, however.

This large parameter space is typically taken as an argument that the images are unbiased when compared to the real world. By this logic, object classification algorithms that work well on the ImageNet or PASCAL VOC datasets ought to perform well on arbitrary images collected from the natural world. In practice, however, this argument does not hold up well. Hoiem et al. [9] made a critical observation along these lines: occlusions do not appear very frequently in the PASCAL VOC data. This observation about PASCAL contradicts common experience. Humans and other visual creatures are quite adept at recognizing occluded objects and navigating through occluded environments because such conditions are normal, not exceptional. This is an example of observer bias. Other authors have noted additional sources of bias, such as predictive statistics from the image background [10, 11]. Such contextual information typically helps prime a classifier towards the correct class of an object. When developing a classification algorithm, however, contextual priming acts as a confounding variable.

The NYU Object Recognition Benchmark (NORB) dataset is a recent example of a* parametric* object classification dataset. This dataset takes a more restrictive approach than ImageNet or PASCAL. Rather than sampling from a large image population with unknown parameters and poorly understood bias, NORB is defined by an explicit parameter space. The point in the parameter space describes the image completely, such that the image could be reconstructed perfectly given knowledge of the parameters. Such an approach is less “natural” in that the images are heavily constrained and look less like scenes from the real world. This follows from the fact that the set of parameters is vastly smaller than the set of parameters needed to describe natural images. The benefit, however, is that the creator of the dataset gets to* choose* which parameters to include. This means that each exemplar in the dataset includes precise, complete, parametric metadata for the design parameters. Such metadata is a necessary condition for constructing experiments to analyze the conditions under which a classification algorithm does well or poorly.

NORB was designed to tackle pose- and lighting-invariant object classification. This goal led to the choice of the following design parameters:

1. Object class.
2. Object instance.
3. Camera elevation.
4. Camera azimuth.
5. Lighting condition.

The* object class* is the category to which the object belongs. NORB contains five categories: four-legged animals, human figures, airplanes, trucks, and cars. The* object instance* is the specific object within the category. Each category contains ten unique instances, for a total of fifty objects. The* camera elevation* and* camera azimuth* describe the position of the cameras relative to the target object. NORB contains images captured from nine elevations and eighteen azimuths. Finally, the* light level* describes the degree of illumination. NORB contains images shot at five different light levels.

These five parameters describe the desired dimensions of variation in the NORB images. For all other possible image properties, the authors worked to carefully control variation. The target objects were painted a uniform color and photographed on a uniform background. The camera and lights were all positioned by robot. The object and cameras were carefully positioned such that the object appears in the center of the frame for both. In practice, this means the five desired sources of parametric variation describe essentially all of the variability in the images.

The tightly controlled variability and high-quality parametric metadata in NORB enable a set of experiments that are not practical with nonparametric datasets like ImageNet and PASCAL VOC. Rotation invariance is an example from this set. Addressing rotational invariance with NORB would simply require slicing the data into appropriate testing and training sets. The training set, for instance, might contain half of the stereo pairs collected from a single camera elevation and azimuth. The testing set would contain all the remaining data. The rich metadata contained in NORB means such a slicing would be easy to construct, and the output would be sufficient to investigate object classification performance as a function of rotation.

Replicating this experiment using the ImageNet or PASCAL VOC datasets would be both significantly more expensive and significantly more difficult. Hoiem et al. [9] offer a template for how to construct the experiment. For a selected subset of the data, it would be necessary to manually label each image with a pose vector. Once each image has a pose vector, it would be possible to slice the data and run an analysis. Experimental control, however, would still be difficult. ImageNet and PASCAL VOC do not contain multiple poses of the same object. This means the training and testing sets would contain nonoverlapping sets of objects. It thus would not be possible to establish how well an object classification algorithm performs on a single object at various degrees of deflection.

The Hoiem et al. [9] study did not attempt to address rotation invariance. It faced a similar problem when attempting to diagnose performance under occlusion, however. The authors had to manually label the level of occlusion for each of the images in the dataset. The source dataset (PASCAL VOC) contained many types and levels of occlusions, so the authors asked the human labelers to classify each object as no, low, medium, or high occlusion. Such an approach is sufficient to establish a relationship between occlusion level and classification performance. It is not sufficient, however, to measure the impact of different types of occlusions or to experiment with strategies to recover lost performance.

*The SORBO Dataset*. The Synthetic Object Recognition Benchmark with Occlusions (SORBO) dataset is a new derivative of the NORB dataset. Like NORB, it is a tightly controlled, parametric dataset. The SORBO dataset applies the same design principles expressed in NORB to the problem of occlusion-resistant object classification. To do this, it includes two image parameters beyond those contained in NORB:* occlusion type* and* occlusion level*. The occlusion type indicates the class of occluding object in a given image. SORBO includes bars, blobs, and random noise occlusion classes. The occlusion level indicates the number of occluded pixels. A pixel does not need to contain an object to be included in this count, so the number of occluded pixels is equivalent to a ratio of occluded pixels over the entire image.

Beyond these two additions, SORBO stays as close as possible to NORB such that NORB results in the literature are directly comparable to SORBO results. In practice, SORBO achieves this aim by extending the NORB data. Rather than starting from entirely new images, SORBO contains NORB images with artificially overlaid occlusions. This is the* synthetic* aspect of SORBO.

The SORBO dataset is intended to address two questions:

1. How do object classification algorithms perform as a function of occlusion level?
2. Are strategies to mitigate the impact of occlusions on classification performance effective?

The first point was covered by the analysis in Hoiem et al. [9]: existing state-of-the-art object classification algorithms are less effective as occlusion increases and nearly useless in heavily occluded conditions. SORBO includes sufficient metadata to replicate this analysis. The second point is uniquely addressable with SORBO. The SORBO dataset includes ground-truth segmentations for all images. This ground-truth segmentation contains an alpha-matte consistent with the approaches in Grady et al. [15] and Rother et al. [16]. Pixel values in the alpha-matte are either 0 or 1, where 0 is entirely background and 1 is entirely foreground.

**Figure 2:**
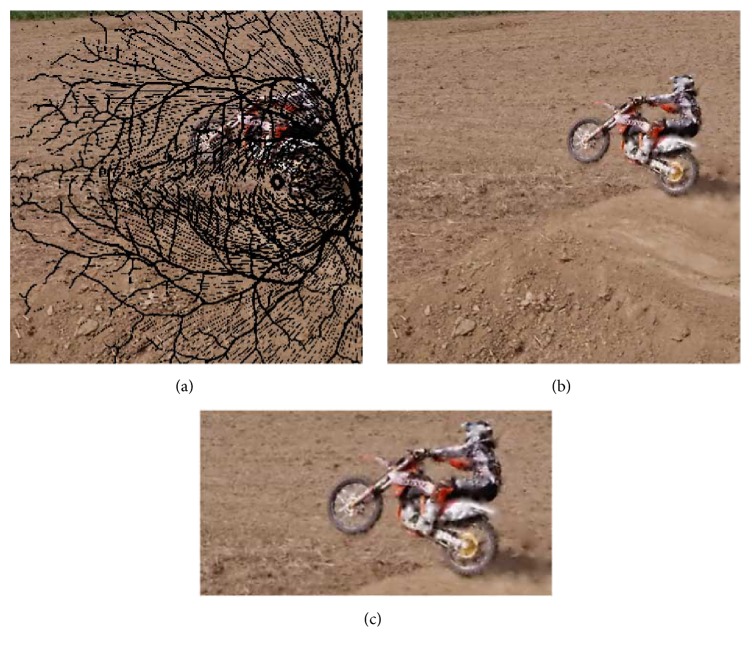
Inpainting over occlusions. The raw input (a) is a frame of video masked with an image of retinal veins. The algorithm does not have access to the pixels covered by the mask. A fast linear diffusion solver (b) uses the raw input and a segmentation estimate to fill in over the occluded regions. Magnifying the inpainted output (c) reveals the weaknesses of this simple technique. The dirt to the left of the image contains little edge information and inpainting works well. The boundaries of the rider, however, are badly blurred where an occlusion is present. More sophisticated inpainting algorithms do not have this problem but at a cost of significant additional complexity and a much longer compute time per frame.

*Inpainting over Occlusions*.* Inpainting* is a standard technique for restoring damaged paintings and images. Originally an exclusively manual process performed by skilled artists to restore art, modern computing has enabled a number of digital equivalents. The essential goal of inpainting is to minimize the appearance of damaged sections by painting over those sections in a manner consistent with the rest of the image [17].  Figure 2 is a successful example.

Automated inpainting algorithms differ primarily on how they define* consistent*. The images in Figure 2 use an algorithm described by Warren [18]. This algorithm models texture as a single color value and consistency as local similarity of color. Pixels that are known to be good are treated as* Dirichlet* boundary constraints. A Dirichlet pixel is one with a* fixed value*. These pixels do not change during diffusion and source color to adjacent floating pixels. After many iterations of color diffusion, floating regions are filled in with information from the surrounding Dirichlet pixels. As shown in Figure 2, this strategy is highly effective when the region to fill in is either relatively small or located in a neighborhood without many edges.

Linear diffusion with boundary constraints tends to produce poor results when filling in large regions or regions that ought to contain many edges. This weakness is primarily a result of the limited texture model. The algorithm is modeling texture as a single color value, but a single color value cannot represent an edge. This means that the algorithm will not propagate edges across large regions of missing information. More sophisticated inpainting algorithms use richer representations to prevent smearing in these large regions. Linear diffusion is substantially more resource-efficient, however. Better algorithms produce better results. Linear diffusion produces good results but requires very little compute time per image. This paper makes no claim about the relative performance of difference inpainting algorithms. Linear diffusion is good enough to demonstrate the key point about classification performance with occlusion, however, and does so at significantly lower computational expense than higher quality algorithms.

## 2. Results

**Figure 3:**
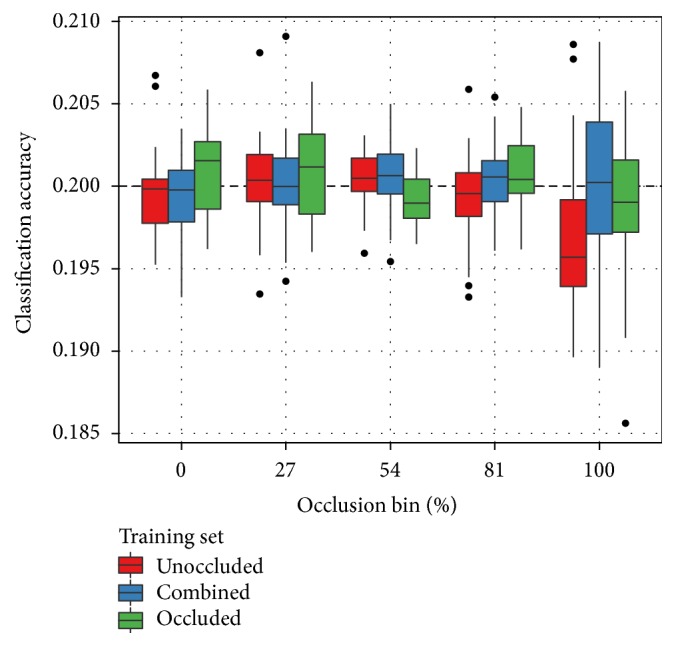
Classification results on SORBO as a function of occlusion level and training set with the chance algorithm. Points outside the box plots indicate outliers. The chance classification algorithm ignores the training data and makes a random guess for each testing sample. SORBO contains five classes with an approximately equal number of examples in each. The expected chance performance is therefore 20%. The occlusion bin percentage indicates the upper bound, inclusive, of the bin. For example, the 0% bin contains testing samples with zero occluded pixels and the 27% bin contains samples with greater than 0% and less than or equal to 27% occluded pixels. As expected, the chance algorithm scores an accuracy of approximately 20% for all trials, at every occlusion level and with every variant of the SORBO training set.

SORBO contains five classes of objects. Each class contains an approximately equal number of examples. This means that a classification algorithm which makes a random guess for each testing sample should still achieve an accuracy of approximately 20%. As shown in Figure 3, this holds up in practice. The *chance* classification algorithm ignores the training data and generates a uniformly random category label for each testing example. This produces accurate predictions of approximately 20% for every trial, at every level of occlusion, and with every variant of the SORBO training set.

**Figure 4:**
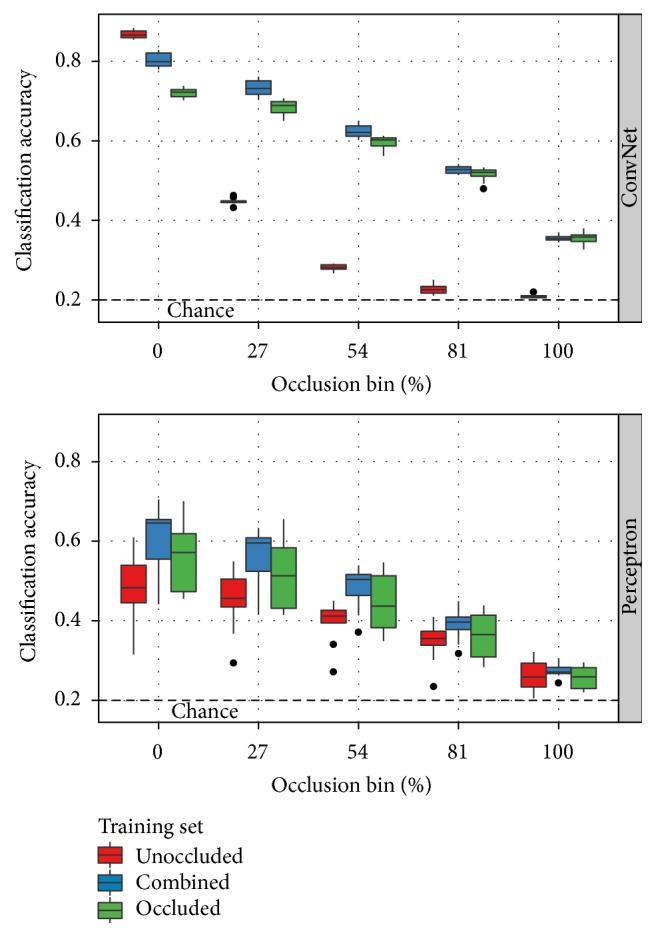
Classification results on SORBO as a function of occlusion level and training set with the Perceptron and ConvNet algorithms. The occlusion bins partition the testing samples as defined in Figure 3. Results with the combined training set are either indistinguishable or better than the corresponding results with the unoccluded or occluded training sets with only one exception. A ConvNet training on unoccluded data outperforms the other two training options when tested on unoccluded data. Performance degrades more rapidly than the other conditions as the level of occlusion in the testing images increases, however.

With the remaining classification algorithms, the training protocol has a significant impact on performance. As shown in Figure 4, the combined variant of the SORBO training set produces the most reliable results. This variant includes both occluded and unoccluded training examples. The unoccluded training set includes only the original 24,300 NORB stereo pairs. While this training condition produces better results with a ConvNet when tested on only unoccluded testing images, the classification performance degrades much more quickly than either of the training conditions that include occlusions. The combined and occluded training set variations include 243,000 examples. Each class of occlusion appears with equal probability. This means the occluded set contains 60,750 more occluded examples than the combined set. The “chance” line indicates the 20% classification accuracy achievable by guessing randomly for each testing example.

**Figure 5:**
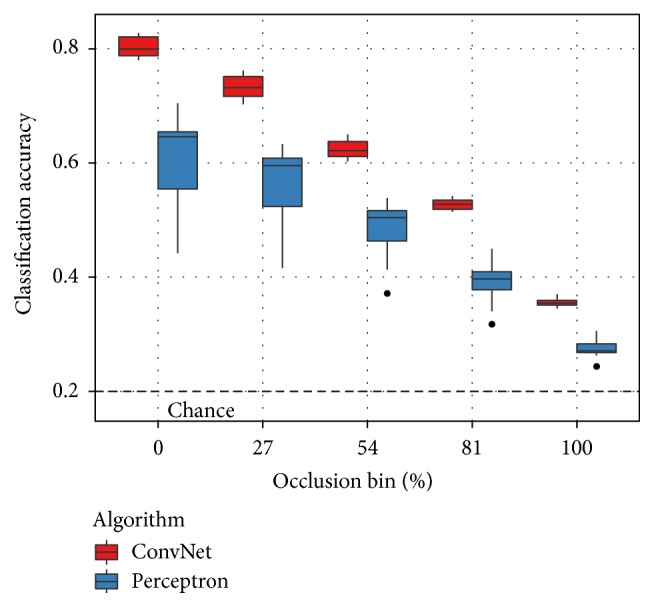
Classification results on SORBO as a function of occlusion level and classification algorithm with the combined training set. The occlusion bins partition the testing samples as defined in Figure 3. Consistent with the analysis of Hoiem et al. [9], classification performance decreases with increasing occlusion and drops to a level marginally better than chance in high-occlusion conditions.

Figure 5 casts these results by classification algorithm rather than training regime. Only the combined training results are shown for clarity. This plot confirms the central result of Hoiem et al. [9] with regard to occlusion: state-of-the-art classification algorithms lose performance as occlusion increases and are only marginally better than chance in high-occlusion scenarios. Both the perceptron and convolutional network classifiers exhibit the same decline. The perceptron classifier, however, performs significantly worse in all conditions. It is also substantially more vulnerable to training order effects. The variance from run to run is much larger than with the convolutional network.

**Figure 6:**
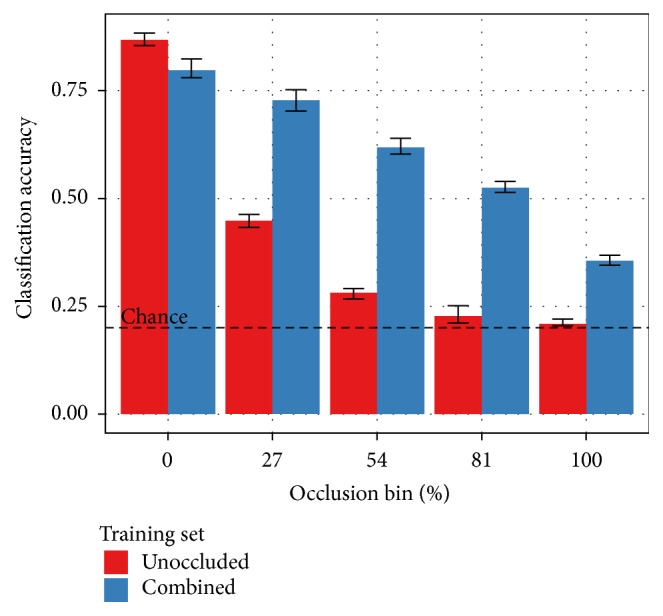
ConvNet classification accuracy when training with either unoccluded or occluded data and no recovery. Both convolutional networks have identical architecture. Both conditions pass the input images to the classifier with no attempt to discount occlusion pixels. In the “unoccluded” case, the network is trained with the 24,300 image pairs in the NORB-simple training set. In the “combined” case, the network is trained with the 243,000 image pairs contained in the SORBO-combined training set. Approximately a quarter of these image pairs are unoccluded. The remaining pairs contain various classes and levels of occlusion. Training with unoccluded images produces higher accuracy on the unoccluded testing images. The performance degrades quickly towards chance as the level of occlusion in the testing images increases, however. The network trained on combined data is less effective on unoccluded images but more robust to increasing occlusion.

All remaining experiments exclude the perceptron classifier.  Figure 6 covers the two no-recovery conditions with the convolutional network classifier. These are the baseline conditions for the construction of an occlusion-robust classifier. In both conditions, increasing occlusion causes a decrease in performance. Training with occluded data reduces the magnitude of this effect and increases robustness. Performance on unoccluded test images decreases, however.

**Figure 7:**
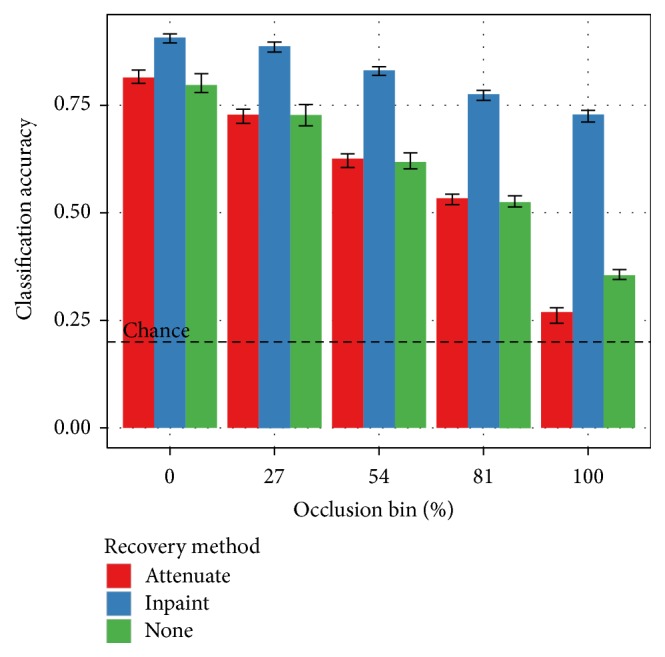
Performance comparison of mechanisms for discounting occluded pixels. All three cases use a convolutional network of identical architecture and trained on the SORBO-combined dataset. Both mechanisms for discounting occluded pixels use the ground-truth segmentation provided with the image data. The discounting mechanism is used during both the training and testing phases. In the “none” case, the training and testing images are passed through to the classifier with no attempt to discount occlusions. In the “attenuate” case, occlusion pixels are set to black before going to the classifier. In the “inpaint” case, occlusion pixels are filled in using a digital inpainting procedure. With a convolutional network classifier, attenuation is worse than the unmodified data. Inpainting, however, is dramatically more effective than the other candidates at every level of occlusion.

Figure 7 builds from the combined case in Figure 6 and adds recovery mechanisms. Performance with the attenuation mechanism is indistinguishable from the no-recovery case at most levels of occlusion. At high levels of occlusion, attenuation is worse than no recovery. Inpainting, however, outperforms the no-recovery case at* all* levels of occlusion. This includes unoccluded test images, suggesting that the disoccluded training images improve generalization.

**Figure 8:**
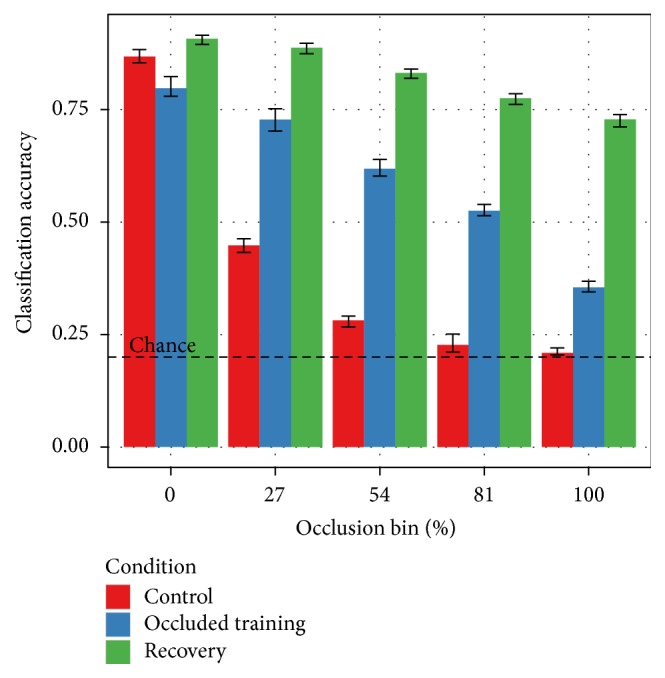
Inpainting improves performance on both occluded and unoccluded test images. The “control” and “occluded training” conditions are the unoccluded and combined training conditions from Figure 6. The “recovery” condition is the inpainting result from Figure 7. The “recovery” condition is consistently better as occlusion increases. Discounting occluded pixels using inpainting has the unexpected benefit of* also increasing performance on unoccluded images*. This is a dataset augmentation effect. The training set in the “recovery” and “occluded training” conditions is ten times larger than in the “control” condition. Inpainting allows the network to leverage this larger training set without overfitting.

Figure 8 further explores the generalization effect. The control and occluded training conditions come from Figure 6. The recovery condition is the inpainting case from Figure 7. Inpainting outperformed occluded training on unoccluded test data. Unoccluded training, however, also outperformed occluded training on unoccluded test data. Figure 8 shows that the recovery condition performs the best even in a direct comparison with the control.

**Figure 9:**
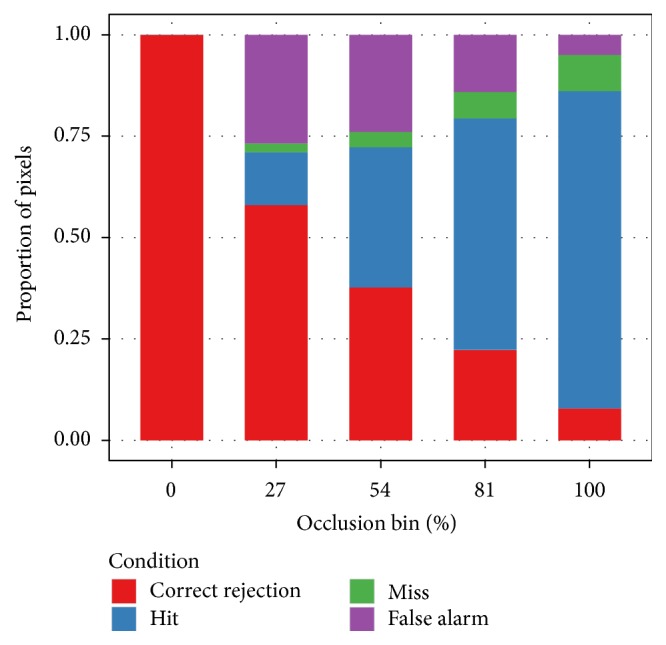
Analysis of segmentation errors. The automated segmentation algorithm leverages the planar structure of the occlusions to estimate which pixels are occlusion and which pixels are figure. Each pixel falls into one of four conditions. In the “hit” and “correct rejection” cases, the estimate is correct. A hit occurs when the algorithm predicts the presence of an occlusion and an occlusion is actually present. A correct rejection occurs when the algorithm accurately predicts the lack of an occlusion. The “miss” and “false alarm” cases are both errors. A miss occurs when the algorithm predicts the lack of an occlusion, but an occlusion is present. A false alarm occurs when the algorithm predicts an occlusion, but no occlusion is present. The accuracy of the automated segmentation process varies depending on the level of occlusion in the image pair. For unoccluded image pairs, the process is entirely accurate. At higher levels of occlusion, overall accuracy drops. False alarms, however, are much more common than misses. This indicates a bias towards marking a given pixel as an occlusion.

All prior experiments used the ground-truth segmentation masks. As suggested by Figure 12 the automated segmentation algorithm does not have perfect accuracy and thus may degrade performance of the classifier.  Figure 9 quantifies the type and level of error. The automated segmentation algorithm makes no errors on unoccluded images. Error peaks at a low but nonzero degree of occlusion. False alarms are more common than misses. Error decreases and the balance of false alarms to misses shifts towards misses as the occlusion level increases. These facts suggest that the algorithm has a bias towards indicating a pixel as an occlusion.

**Figure 10:**
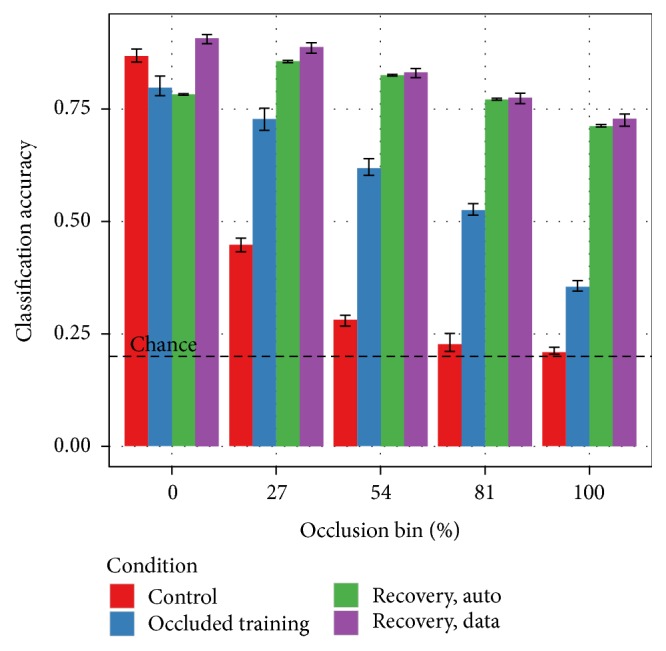
Performance with inferred versus ground-truth segmentation. The “control,” “occluded training,” and “recovery, data” conditions are the same as in Figure 9. The “recovery, auto” condition is new and matches the “recovery, data” condition except for the source of the segmentation masks. The data case uses the ground-truth segmentations provided with the dataset. The automatic case uses only the raw stereo image pairs and infers the segmentation masks. These two recovery conditions perform at parity at higher levels of occlusion. Using inferred segmentations erases the dataset augmentation effect observed in Figure 9, however. Performance on unoccluded test images is no better than the occluded training condition.

Figure 10 expands Figure 8 with a second recovery condition. The second recovery condition uses the automated recovery algorithm rather than the ground-truth segmentations. Performance with the new automated segmentation algorithm matches the system using ground-truth segmentations in high-occlusion conditions. The generalization effect on unoccluded testing images no longer appears, however.

## 3. Discussion

Objects that are heavily occluded are more difficult to classify than unobstructed objects. Pervasive observer bias in the major object classification datasets has masked this effect, however, limiting the utility of state-of-the-art object classification algorithms in the real world.

This paper introduced the Synthetic Object Recognition Benchmark with Occlusions (SORBO) dataset. SORBO is a derivative of the earlier NYU Object Recognition Benchmark (NORB). Like NORB, SORBO is* parametric* and optimized for detailed analysis of classification performance. SORBO adds various classes and levels of stereo occlusions to NORB images to enable precise measurement of classification performance as a function of occlusion. The dataset is paired with an infrastructure suitable for high-throughput experimentation on a compute cluster.

Results on SORBO reproduce the analysis in Hoiem et al. [9]. A convolutional neural network exhibits high performance on unoccluded testing data but degrades rapidly with increasing occlusion. At the highest level of occlusion, the performance of a high-quality classifier is little better than random chance. Training the classifier with a mix of occluded and unoccluded images produces the most reliably good results. All training conditions yield poor performance on highly occluded test images, however.

Augmenting a high-quality classifier with an inpainting preprocessing stage successfully recovers much of the lost performance. Inpainting using either a ground-truth or automatically extracted segmentation mask preserves the majority of performance all the way up to the highest level of occlusion. These results suggest that occlusion-robust classification is possible so long as the problem of splitting the input into figure, ground, and occlusion is solvable.

While the automatically extracted segmentation masks are fully accurate for unoccluded images, recovery using the ground-truth masks indicates a surprising gain in performance. The ground-truth recovery condition outperforms both the control and occluded training conditions for unoccluded test images. This gain suggests the inpainting with ground-truth segmentation masks reduces overfitting and improves generalization.

The results suggest two key findings. First, occluding and then disoccluding a dataset using inpainting is a viable technique for dataset augmentation. State-of-the-art classification systems typically include one or more augmentation techniques to increase the size of the training dataset, reduce overfitting, and improve generalization. Reflections, linear shifts, and elastic distortions are three common classes of augmentation applied to object classification problems. All three build an invariance to an expected transformation in the data. The digit three is still a three when shifting several pixels to the left, for instance. The SORBO results indicate that occlusion is another useful class of dataset augmentation.

Second, extending a standard classification algorithm with an inpainting preprocessing stage produces a system that performs at parity with the base system on unoccluded test images but exhibits substantially more robust performance as occlusion increases. The critical variable for real-world systems is the quality of the segmentation masks. A classifier working in concert with a high-quality segmentation algorithm can perform nearly as well on heavily occluded images as unoccluded images.

## 4. Materials and Methods

The principle technical objectives for this work are to reproduce the results from Hoiem et al. [9] as a baseline case and enable experiments in occlusion-resistant object classification algorithms. These goals necessitate a scalable experimental infrastructure that supports large parametric studies.

In this case,* scalable* means that the data collection infrastructure can efficiently distribute work over a pool of compute nodes. Given a perfectly efficient distribution of work, a task that would take a hundred hours to execute on a single compute node would require only four hours to execute on a cluster of 25 compute nodes. Most compute tasks cannot be distributed this efficiently over a large pool of compute nodes. The overhead required to keep all of the compute nodes synchronized limits performance as the size of the cluster increases. In this case, however, each trial is* independent*. The compute nodes do not require synchronization during the trials. This is an example of an* embarrassingly parallel* job, for which the theoretical scalability is nearly perfect. Given the large volume of work and the embarrassingly parallel nature of the problem, an experimental infrastructure designed to operate on a compute cluster is a reasonable investment.

All SORBO experiments used a two-tier parallel infrastructure. The management tier consists of a* version control server* and a* head node*. The compute tier contains 16* compute nodes*. The version control server is responsible for storing the source code and small binary assets, as well as a history of changes. The rest of the infrastructure only executes code checked out from the version control server to ensure that all simulations are traceable and repeatable. The head node is responsible for storing large binary assets, coordinating experiments, distributing work to the compute nodes, and archiving the results. Seed data for SORBO is stored here because it is too large to place on the version control server. The compute nodes are responsible for the actual execution of trials.

Validating the Hoiem et al. [9] study required three classifiers and three types of training data. The three classifier types are* chance*,* perceptron*, and* ConvNet*. The three types of training data are* unoccluded*,* occluded*, and* combined*.

The chance classifier simply discards the training data and generates random predications for the testing data. The perceptron classifier is a linear network trained by stochastic gradient descent. The ConvNet classifier is a multilayer convolutional neural network modeled after the classifier in LeCun et al. [12].  Section 4.2 contains additional details on these algorithms.

Each of the training data conditions is a different slice of the SORBO dataset. The unoccluded training condition contains only NORB images, so algorithms trained in this condition are directly comparable to NORB results in the literature. The occluded condition contains only images with occlusions applied. The mixed training data condition contains both types of data.  Section 4.2 contains additional details on each condition.

### 4.1. SORBO Construction

SORBO derives from the NORB normalized-uniform dataset, also termed “small NORB.” Like NORB, it contains training and test sets of 96 × 96 pixel stereo images shot with fixed disparity. Also like NORB, SORBO contains descriptive metadata for the object class, object instance, camera elevation, camera azimuth, and light level for each image. SORBO adds two features to NORB:

1. Descriptive metadata on the class and level of occlusion.
2. A ground-truth segmentation for each image pair.

The additional metadata contains sufficient information to completely describe the occlusion composited over each image. Every image contains two new metadata fields: *occlusion*_*type* and *occluded*_*pixels*. The occlusion type field is a factor variable with a value chosen from the set {*none*,* random*,* bars*,* blobs*}. The occluded pixels field contains an integer count of occlusion pixels summed across the left and right images. The count is taken over* every pixel in the field of view*, not just the pixels where the target object is present. This means the maximum value for *occluded*_*pixels* is 2*∗*96*∗*96 or 18432. The underlying assumption for this choice is that all pixels from the normalized-uniform NORB data are considered figure. The target object in the data is of normalized size and centered on a uniform background, so this is a reasonable assumption.

Additional metadata fields describe the specific occlusion in each image for every occlusion type except *none*. For occlusion type *random*, there are supplementary *seed* and *threshold* fields. The seed is an integer used to prime the pseudorandom number generator that produces the random occlusion. The threshold field is a floating-point value in the range [0,1) that controls the fraction of pixels that are occluded.

For occlusion type *bars*, there are supplementary* omega*, *theta*, *phase*, and *threshold* fields that control the spacing, orientation, and thickness of the occluding noise bars. Bar occlusions are generated by thresholding a sinusoidal function along an offset axis. Omega is the angular frequency of the sinusoid. Theta is the angular offset with respect to the horizontal. The phase determines the starting point on the sinusoidal function. The threshold is a floating-point value in the range [0.1,0.9). Like the random case, it controls the percentage of pixels that are occluded.

For occlusion type *blobs*, there are supplementary *seed*, *blob*_*n*, and *blob*_*scale* fields. Like the random case, the seed is an integer used to prime a pseudorandom number generator. The *blob*_*n* field is an integer describing the number of blobs in the image. The *blob*_*scale* field is an integer describing the size of the blobs.

The *occlusion*_*type* field, along with the supplementary fields, fully describes the occlusions and is sufficient to reconstruct them given an additional disparity parameter. The disparity parameter is an integer that describes the ocular disparity of the stereo occlusions measured in pixels. It is a global parameter with a value of five. This value places the occlusions at a depth closer to the observer than the target objects. It is an integer to simplify segmentation. With an integer value, every pixel in the data contains information for either the background or the occlusion with no mixing. There are no border pixels that contain some information from the target object and some from the occlusion layer. A natural photograph would very rarely have objects fall precisely on pixel boundaries in this way.

Beyond the additional metadata, SORBO also contains ground-truth segmentations for both the training and testing sets. These ground-truth segmentations adopt the alpha-matte standard used in Grady et al. [15] and Rother et al. [16]. For each stereo pair in the data, SORBO contains an additional pair of 96*∗*96 binary alpha-matte images. A value of 1 in the alpha-matte indicates that the pixel is an occlusion. A value of 0 indicates the lack of occlusion.

Like NORB, the images in SORBO are grayscale. For compact storage, each pixel in NORB is described by a single byte. This means the images in NORB contain 256 gray levels and each pixel has a value in the range [0,255]. Zero is black and 255 is white. For SORBO, ease of integration with off-the-shelf object classification algorithms was a higher priority than compactness of representation. SORBO is stored as 32-bit floating-point values in the range [0,1], where 0 is black and 1 is white. This choice of simplicity over compactness is reasonable given that networks are much faster and disks are much larger than those available when NORB was created.

Occlusions in SORBO are textured with binary noise. Each occluded pixel has a value of either 0 or 1, chosen from a uniform distribution. This texture is necessary to both maximally confuse the object classification algorithms and provide an information source for the performance-recovery algorithms. See Section 4.4 for more details on this choice.

SORBO contains three different training sets and one test set. The first training set, called *clean*, contains only the 24,300 original images from the NORB normalized-uniform training set. No occlusions are added. This training set is a baseline that allows direct comparison with the many NORB results in the literature.

The second and third training sets are called *comb*, or combined, and *occ*, or occluded. Both training sets contain 243,000 stereo pairs. This size is consistent with the jittered and cluttered versions of the NORB dataset. Each stereo pair is generated by selecting a random stereo pair (with replacement) from the 24,300 pairs in the NORB normalized-uniform training set, selecting a random occlusion type, generating random parameters for the chosen occlusion type, and generating random noise to serve as the texture for the occlusion. The combined training set contains stereo pairs with all four occlusion types, including *none*. The occluded training set excludes the *none* occlusion type, so all stereo pairs are occluded. All random choices are made with a uniform distribution over the possible options.

The *none* occlusion type has no supplemental metadata, so no additional random choices need to be made and no occlusion texture is generated. The *random* occlusion type requires a seed and a threshold. The seed is generated from the space of all possible 32-bit integers. The threshold is floating-point and generated from the range [0,1). The *bars* occlusion type requires floating-point omega, theta, phase, and threshold parameters. Omega is generated from the range [0.5,10), theta from [0, *π*), and phase from [0, *π*). The threshold is generated from the range [0.1,0.9). Finally, the *blobs* occlusion type requires *seed*, *blob*_*n*, and *blob*_*scale* parameters. The seed is generated from the space of all possible 32-bit integers. The *blob*_*n* parameter is an integer generated from the range [1,9]. The *blob*_*scale* parameter is an integer generated from the range [−5,5].

The SORBO testing set is generated using the same procedure as the combined training set, with two exceptions. First, the SORBO testing set uses the NORB* testing* stereo pairs instead of the training stereo pairs. The NORB testing and training sets are structured identically. The difference is the object instances present in each set. The training data contains instances 4, 6, 7, 8, and 9 of each category. The testing data contains the remaining instances. This means the testing and training data do not contain the same objects, just the same object categories. Second, the SORBO testing set only contains 97,200 stereo pairs. This dataset size is consistent with the jittered and cluttered versions of NORB.

#### 4.1.1. SORBO Construction Procedure

The full SORBO dataset contains 607,500 grayscale stereo pairs and an equal number of alpha-mattes stored as 32-bit floating-point values, for a total of approximately 83 gigabytes of raw data. A total of 64 bits are consumed to represent each pixel, including both the gray level and alpha-matte. Reducing the per-pixel data from 64 bits to 9 bits, including 8 for the gray level and 1 for the binary alpha-matte, would reduce the raw data volume to approximately 12 gigabytes. Compression might reduce this value by another order of magnitude.

The raw data volume, however, is not the limiting factor for performance. Good machine learning practice dictates that the training data should be shuffled every time it is used to train a classification algorithm. If the SORBO image data were precomputed and placed on disk, each training run would need to seek randomly in the data file for each stereo pair. Disk seeks are very expensive. Reading large blocks of sequential data from a hard disk is substantially more efficient than reading small blocks of random data.

Spreading work across a large compute cluster raises an additional set of practical issues. The data has to be physically placed on one or more disks. Placing it on a single disk and allowing the remaining nodes to access that master copy over the network is simpler, but the number of disk seeks per training epoch per disk increases linearly with the number of compute nodes and the network limits performance. This is* disk seek amplification*. Copying the data to a scratch disk on each node requires additional checks to ensure that stale versions of the data are expunged and each node always has a fresh copy. The number of disk seeks per training epoch per disk remains constant, however, so throughput is dramatically higher.

Leveraging the detailed occlusion metadata, the SORBO construction process uses a third option. The NORB normalized-uniform testing and training image data lives on a single node with a shared file system, along with the full SORBO metadata. This metadata is vastly smaller than the image data, at only tens of megabytes. The NORB normalized-uniform image data is vastly smaller than the full image data for SORBO, at approximately 3 gigabytes when stored in 32-bit floating-point format.

When a compute node needs to construct a shuffled version of SORBO to train or test a classification algorithm, it reads the metadata from the shared filesystem,* shuffles only the metadata*, and builds a local, shuffled copy of the SORBO image data on the fly. For each row in the shuffled metadata, the compute node fetches the appropriate image from the NORB files on the shared filesystem, adds the appropriate occlusion, and writes a copy of both the occluded data and the alpha-matte to the local disk.

Superficially, this scheme appears to suffer from the same disk seek amplification problem as the simpler scheme in which SORBO is precomputed and stored on a single shared filesystem. In practice, however, only approximately 1.5 gigabytes of data is “hot” at any given time, meaning being read by the compute nodes. This is a small enough volume that the operating system caches on the node hosting the shared filesystem can absorb the load. Given the interconnect and node properties of the compute cluster, reading the NORB data from a single node does not adversely affect total performance.

This hybrid scheme splits the construction of SORBO into two phases. The first phase is a preprocessing stage to prepare the metadata and convert the NORB normalized-uniform image sets to a suitable format. A single compute node executes this stage, since it only needs to occur once. The compute node writes the metadata to the shared file system as a textual file in comma-separated values (CSV) format. It also writes the NORB image data as two binary files, one for training and one for testing; each contained a sequence of 32-bit floating-point values in little-endian format. The sequence order is row major, assuming a 24300*∗*2*∗*96*∗*96 array of data. The binary file contains no header or delimiter information. This format is efficient because it directly corresponds to the layout of the data in memory.

In the second stage, the worker compute nodes load the CSV metadata, map the appropriate block of NORB image data in to memory, and build a local, shuffled copy of SORBO on the fly. To keep memory usage under control and accelerate the simulations, this process is parallelized over a pool of worker processes on each node. Object classification algorithms do not get access to the entire dataset at once. Instead, they get a sequence of minibatches, each containing a thousand rows of input data. This eliminates the need to wait for the entire dataset to finish processing before the object classification algorithm can start working. As each minibatch is completed, it is passed in to the object classification algorithm.

### 4.2. Benchmark Algorithms and Training

The processing pipeline includes three reference object classification algorithms. The first is a chance classifier that ignores the training data and makes random predictions for the testing data. The second is a simple linear perceptron trained by stochastic gradient descent. The third is a convolutional neural network (ConvNet). Both the linear classifier and ConvNet implementations are off-the-shelf open-source libraries.

The linear classifier comes from the scikit-learn library [19]. Scikit-learn is an open-source machine learning toolkit written for the Python language. SORBO classification results use the “sklearn.linear_model.Perceptron” classification algorithm with default parameters. A perceptron classifier is a good choice because the algorithm is simple, highly scalable, and amenable to incremental training. Perceptrons only require one data point at a time in order to learn. This means the processing pipeline can stream data past the classifier without keeping the entire dataset in memory. The classifier was trained with a single pass through the shuffled training set.

The convolutional network uses the open-source cuda-convnet package [20]. This package offers a highly optimized implementation of convolutional networks for NVIDIA graphics processors. The specific network structure is user-configurable. All SORBO experiments used a two-layer network. The first layer is convolutional and used a bank of 16 5-by-5 stereo filters with a hyperbolic tangent output function. This hyperbolic tangent is followed by an absolute value nonlinearity. An absolute value nonlinearity outperformed a rectifying nonlinearity in pilot experiments. The second layer is a fully connected network with five outputs. These five outputs correspond to the five object classes. The output from the fully connected layer passes through a softmax function to generate final prediction probabilities.

The ConvNet classifier required 60 passes through the full training dataset to converge. For the first 40 passes, the convolutional and fully connected layers used a learning rate of 0.001 on the weights and 0.002 on the bias values. Passes 41–50 used rates of 0.0001 and 0.0002. Passes 51–60 used rates of 0.00001 and 0.00002. The decreasing learning rates are a form of* early stopping* [21]. After 40 passes through the training data, the network starts to overfit when trained with a high learning rate and becomes less accurate. The decreasing learning rate for the final 20 passes allows the network to fine-tune performance without overfitting.

### 4.3. Recovery Using Attenuation or Inpainting

The SORBO pipeline includes two recovery algorithms. The first is a simple attenuation. This algorithm produces a final input image by setting occlusion pixels to black. Figure pixels pass through without modification. Attenuation leaves visible occlusions but eliminates the high-frequency texture on the occlusions.

**Figure 11:**
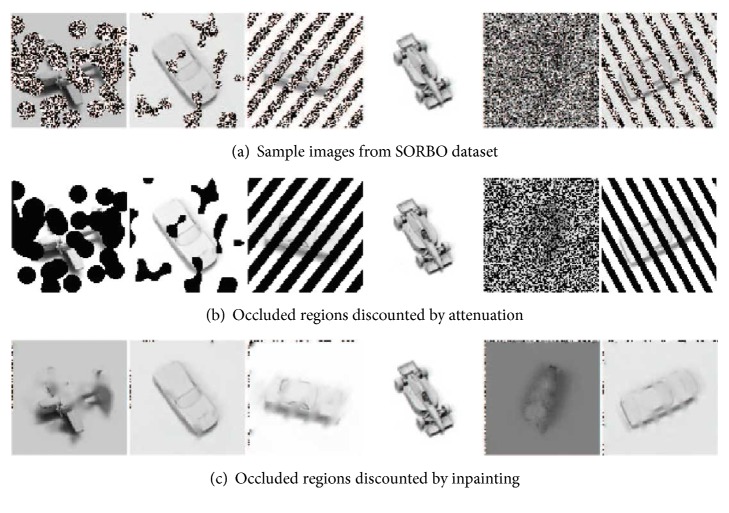
Occlusion recovery using attenuation and inpainting. Sample images from the SORBO dataset (a) are recovered using either attenuation (b) or inpainting (c). These recovery methods are candidate techniques for* discounting irrelevant information* from the images. Attenuation removes the occluding texture by setting occluding pixels to black. Inpainting infers a plausible value for the occluded pixels by using the remaining visible pixels. The dots along the borders of the inpainted images are an artifact of a boundary condition bug in the inpainting library routine.

The second recovery algorithm is an open-source digital inpainting algorithm from the OpenCV library. This algorithm treats occluded pixels as damage and attempts to fill them in using information from neighboring pixels. The result is an image with no visible occlusions, but varying amounts of distortion due to the missing information at the locations of the occlusions.  Figure 11 contrasts these two strategies.

### 4.4. Stereo Segmentation

Automatic segmentation is a difficult and frequently ambiguous problem. State-of-the-art segmentation algorithms like GrabCut still require input from a human to cue the algorithm to the correct target object and clean up the results [16].

In practice, the stereo occlusions in SORBO are easy to segment. This follows from the orientation of the occlusions relative to the observer. SORBO uses planar occlusions placed perpendicular to the camera. This means that the occlusion portions of the left and right images will match perfectly when the two images are aligned correctly. The automatic segmentation process leverages this property to estimate the locations of the occluding pixels by correlating the left and right input images and looking for a sharp peak in the correspondence. A peak is defined as a value that is twice the magnitude of its immediate neighbors. After identifying a peak in the correspondence, the algorithm predicts a class for each pixel. Pixels that are very similar when the left and right images are aligned are classified as occlusion.

The segmentation algorithm is effectively a simple approximation of stereo depth estimation. Estimating the depth of a pixel from a stereo image pair requires solving a* correspondence* problem between the left and right images. For a given landmark in the environment, the correspondence problem is to locate that landmark in both the left and right images from the stereo pair. These two pixel-space coordinates, along with the distance between the cameras when taking the stereo pair, are sufficient to solve for the distance to that landmark. The planar, perpendicular, textured nature of the occlusions yields a very simple correspondence problem. Occlusions are simply pixels in the near depth plane.

**Figure 12:**
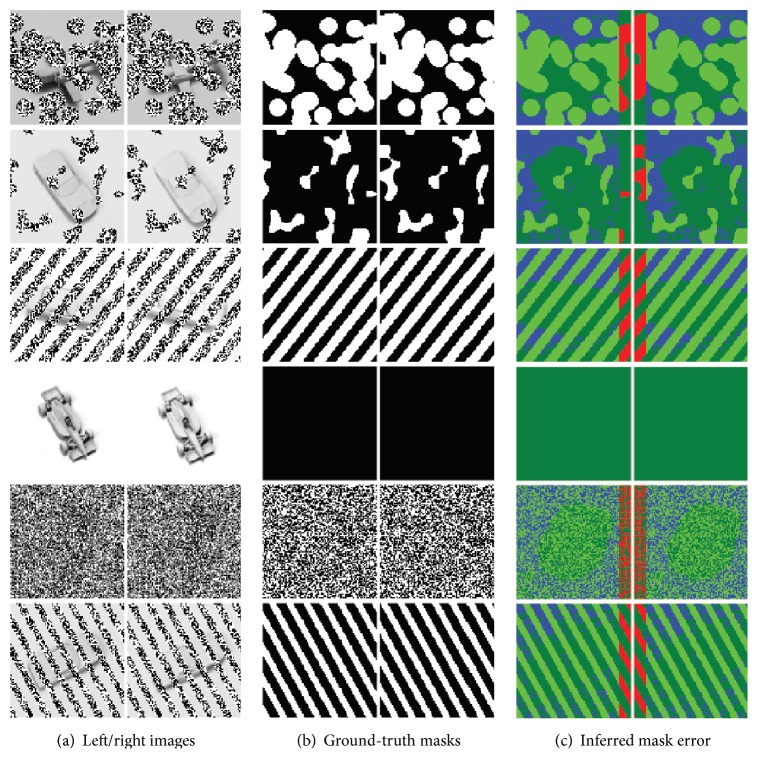
Accuracy of automatic stereo segmentation. NORB and SORBO are stereo datasets. Sample images from SORBO (a) contain various types and level of occlusions. SORBO also includes corresponding ground-truth segmentation masks (b). In these masks, white identifies occlusion pixels and black identifies target pixels. The stereo estimation algorithm produces a mask using only the raw input images (a). These estimates tend to have a high false positive rate but miss occlusions only at the borders (c). In these error images, light green corresponds to a hit. Dark green is a correct rejection. Blue is a false prediction of occlusion or false positive. Red is a missed prediction.

Sample outputs from the algorithm are shown in Figure 12.
